# Clinically Informed Automated Assessment of Finger Tapping Videos in Parkinson’s Disease

**DOI:** 10.3390/s23229149

**Published:** 2023-11-13

**Authors:** Tianze Yu, Kye Won Park, Martin J. McKeown, Z. Jane Wang

**Affiliations:** 1 Department of Electrical and Computer Engineering, University of British Columbia, Vancouver, BC V6T 1Z4, Canada; tianzey@ece.ubc.ca; 2 Pacific Parkinson Research Centre, University of British Columbia, Vancouver, BC V6T 1Z4, Canada; karabach88@gmail.com (K.W.P.); martin.mckeown@ubc.ca (M.J.M.); 3 Department of Neurology, Faculty of Medicine, University of British Columbia, Vancouver, BC V6T 1Z4, Canada

**Keywords:** Parkinson’s disease, finger tapping, UDPRS quantification, data-driven, machine learning

## Abstract

The utilization of Artificial Intelligence (AI) for assessing motor performance in Parkinson’s Disease (PD) offers substantial potential, particularly if the results can be integrated into clinical decision-making processes. However, the precise quantification of PD symptoms remains a persistent challenge. The current standard Unified Parkinson’s Disease Rating Scale (UPDRS) and its variations serve as the primary clinical tools for evaluating motor symptoms in PD, but are time-intensive and prone to inter-rater variability. Recent work has applied data-driven machine learning techniques to analyze videos of PD patients performing motor tasks, such as finger tapping, a UPDRS task to assess bradykinesia. However, these methods often use abstract features that are not closely related to clinical experience. In this paper, we introduce a customized machine learning approach for the automated scoring of UPDRS bradykinesia using single-view RGB videos of finger tapping, based on the extraction of detailed features that rigorously conform to the established UPDRS guidelines. We applied the method to 75 videos from 50 PD patients collected in both a laboratory and a realistic clinic environment. The classification performance agreed well with expert assessors, and the features selected by the Decision Tree aligned with clinical knowledge. Our proposed framework was designed to remain relevant amid ongoing patient recruitment and technological progress. The proposed approach incorporates features that closely resonate with clinical reasoning and shows promise for clinical implementation in the foreseeable future.

## 1. Introduction

Parkinson’s Disease (PD) is the most-common neurodegenerative movement disorder characterized by various motor symptoms, including resting tremor, bradykinesia, muscular rigidity, and postural instability [1]. Among these symptoms, bradykinesia, the slowness of movement often associated with a reduced movement amplitude, is a fundamental feature and a key diagnostic criterion for Parkinsonism [2].

The Unified Parkinson’s Disease Rating Scale (UPDRS) is recognized as the definitive standard for assessing the severity of Parkinson’s Disease (PD). This scale has undergone a partial revision via the Movement Disorder Society (MDS), resulting in the MDS-UPDRS [3]. An essential element of the MDS-UPDRS is the motor examination (Part III), encompassing 18 motor tasks performed by the patient and evaluated by a rater, who scores each task based on the observed level of impairment.

A significant task in this scale, Item 4, is finger tapping, which is used to gauge bradykinesia. In this test, patients are instructed to tap their index finger to their thumb as quickly and widely as possible, completing ten taps. The rater observes and evaluates several aspects of this action, such as the speed and amplitude, any noticeable decrease in speed or amplitude, and the presence of hesitations or stops. Each side of the body is evaluated separately, and scores are assigned on a scale from 0 (normal) to 4 (severe), as outlined in Table 1.

While the transition from the UPDRS to the MDS-UPDRS addressed several uncertainties and shortcomings of the original version, it did not completely overcome all challenges. Notably, the subjective nature of this manually administered clinical scale persists, resulting in potential scoring discrepancies by the same (intra-rater) or different (inter-rater) evaluators. Moreover, human assessments of this nature can be both time-intensive and costly.

Advancements in machine learning technologies have greatly improved the objective and automated assessment of Parkinson’s Disease (PD) [4,5,6,7]. Notably, wearable sensor technology has been utilized to measure bradykinesia during finger tapping [8,9], with several of these sensors now available for clinical use [10,11]. The use of machine learning in the assessment of Parkinsonism holds promise for clinical care, potentially providing a more-efficient, extensive, and remote monitoring system. Additionally, machine learning approaches could greatly benefit clinical trials by offering a quantitative, objective digital biomarker for Parkinsonism.

However, concerns regarding costs and potential issues with patient compliance regarding wearable sensors have led to growing interest in video-based analysis. This method offers a non-contact, unobtrusive alternative for the automated assessment of motor symptoms in PD [12,13]. That said, there are technical challenges associated with the remote interpretation of video recordings [5]. A significant limitation of existing video-based assessments of PD is their focus on only a subset of the clinically relevant features detailed in the UPDRS instructions, often resulting in limited clinical interpretation of the models. Given that UPDRS is grounded in decades of clinical expertise and encompasses a wide range of bradykinesia symptoms in Parkinsonism, both the comprehensiveness and explainability of any model are vital. Furthermore, many studies have relied on solely collecting data in a standardized video collection environment, raising concerns about the sustainability and adaptability of these models. Addressing these gaps, our study aimed to develop a machine-learning model for the automatic prediction of UPDRS bradykinesia scores from finger tapping videos. We focused on enhancing the robustness of our model by extracting features that are in strict accordance with the clinical guidelines provided in the UPDRS, employing a white-box model for better explainability. This approach also involves incorporating a variety of video collection environments to ensure broader applicability and resilience of the model in diverse settings.

## 2. Related Work

In this section, we examine the existing literature on the automated assessment of Parkinson’s Disease (PD) severity using Machine Learning (ML) techniques. The subsequent subsections offer a comprehensive overview of cutting-edge methodologies and their respective contributions to this field.

### 2.1. Advances in Automated Assessment of Parkinson’s Disease with Machine Learning Approach

The majority of ML-based automated assessment of PD approaches utilize body-worn inertial sensors, such as accelerometers and gyroscopes. These sensors include independent devices integrated with short-range communication capabilities [8], as well as sensors embedded in smartphones or smartwatches that facilitate data collection using the patients’ own mobile devices [14]. AI technology using sensor signals has been used to investigate a wide range of PD symptoms, both motor and non-motor, including bradykinesia, tremor, rigidity, gait disturbances, freezing of gait, falls, sweating, and sleep disturbances [8]. Bradykinesia, a key symptom for diagnosing PD, is the most extensively investigated symptom [8]. Various studies exploring bradykinesia, resting tremor, or rigidity in the upper limbs have employed commercially available sensors placed on the arms, wrists, or fingertips, yielding diverse outcome measures [8,15,16]. The use of sensor technology for detailed gait analysis, particularly for measuring parameters like stride length, velocity, and cadence, has been emphasized due to the debilitating nature of gait and balance impairments in PD [17,18]. Additionally, focused research has been conducted on specific PD motor symptoms such as dysarthria and micrographia [19,20,21]. However, there are significant limitations associated with wearable-sensor-based monitoring. First, the need for patients to wear these devices can be intrusive, potentially leading to reduced compliance, particularly in the elderly population, primarily impacted by PD. Additionally, the diverse levels of digital literacy among older adults pose further challenges. Consequently, there have been suggestions for alternative approaches to wearable inertial sensors. Notable among these alternatives are techniques employing electromagnetic signals [22], with video-based assessment also gaining prominence as a major competitor to wearable sensors.

### 2.2. The Current Landscape of Video-Based Assessment for Parkinson’s Disease

Capitalizing on the remarkable advancements in computer vision technology, a multitude of studies have focused on analyzing the motor symptoms of Parkinson’s Disease (PD) using video data. While the precise methodologies vary across these studies, they typically adhere to a four-step process: (i) recording symptoms with a camera, (ii) estimating human poses, (iii) extracting pertinent kinematic features of the motor symptom, and (iv) implementing machine learning models for analysis.

In the realm of video-based Parkinson’s Disease (PD) assessment, studies generally fall into two categories based on their initial video acquisition approach: those employing advanced video recording technologies and those using standard consumer-grade RGB cameras. Advanced technologies include RGB–depth cameras like Microsoft Kinect, which, by providing 3D data indicating the distance between the captured subject and the camera, are used to assess the bradykinesia of the arms or gait impairment in PD [23,24,25].

Examples also encompass innovations like the Leap Motion controller, a virtual reality tool for upper limb rehabilitation in PD patients through serious games [26]. The first-generation system utilized three LEDs and two infrared cameras, capturing data at a resolution of 640×240 at 120 frames per second, designed to function optimally within a 60 cm range. This system is now being integrated into automatic PD classification research [27,28,29]. However, a large portion of research still relies on readily available consumer cameras, such as standard camcorders and smartphone cameras, for daily clinical practice [4,30,31,32]. Regarding the categorization of these studies, they can be either marker-based Motion Capture systems (MoCaps) or markerless, determined by whether or not they use body-attached markers. Infrared MoCaps, while the gold standard for clinical gait analysis, require extensive space and equipment, limiting their accessibility. Recent studies, therefore, are exploring the viability of markerless systems as a feasible alternative to MoCaps [33,34].

For the human pose estimation step, various studies incorporate specialized or generic 3D or 2D pose estimation algorithms. Popular open-source libraries like OpenPose [35], Google MediaPipe [36], and AlphaPose [37] are frequently employed for systems designed to assess PD motor symptoms. Here, we chose MediaPipe for its efficiency, effectiveness in diverse environments, and regular updates by the GoogleAI team.

In terms of kinematic feature extraction and their application in machine learning models, there is a range of utilized features and models. Park et al. [32] extracted kinematic features related to the amplitude, velocity, and decremental response of bradykinesia from 110 videos of finger tapping and utilized a Support Vector Machine (SVM) classifier to show good reliability with clinical ratings. Vignoud et al. [38] proposed a method using seven parameters extracted from finger tapping and hand movement videos representing speed, amplitude, fatigue, and periodicity to predict the appropriate UDPRS score with linear regression and Decision Tree models. Liu et al. [39] incorporated a more-novel network architecture, a Global Temporal-difference Shift Network (GTSN), to estimate the MDS-UPDRS tremor scores from video recordings. A Convolutional Neural Network (CNN) examining location, velocity, and inter-joint relationship information derived from video recordings has also been proposed [40].

It should be noted that, while the Unified Parkinson’s Disease Rating Scale (UPDRS) advises evaluators to consider factors such as amplitude, velocity, decrement, and pauses or hesitations, numerous studies have focused on a limited subset of these attributes. Recognizing this, recent research has increasingly prioritized the extraction of more-comprehensive features and the interpretability of the resulting models. Morinan et al. [4,30] introduced a Random Forest algorithm for classifying bradykinesia, utilizing 11 kinetic features derived from video recordings of 1156 MDS-UPDRS assessments across five medical centers. Similarly, Islam et al. [31] selected clinically pertinent features from an initial set of 53, explaining their model’s performance by employing the Shapley Additive Explanations method, alongside various regression models. Within the extensive array of pertinent research, we have distilled and summarized the four most-current and -relevant studies that align closely with our investigation in Table 2.

In light of the trends identified in previous research, our analysis utilized standard RGB cameras in two different recording environments, combined with MediaPipe for human pose estimation. We fine-tuned our methodology for extracting kinematic features to better correspond with the clinical characteristics of bradykinesia. Furthermore, we opted for a Decision Tree model to improve the interpretability of our findings.

## 3. Method

**Figure 1:**
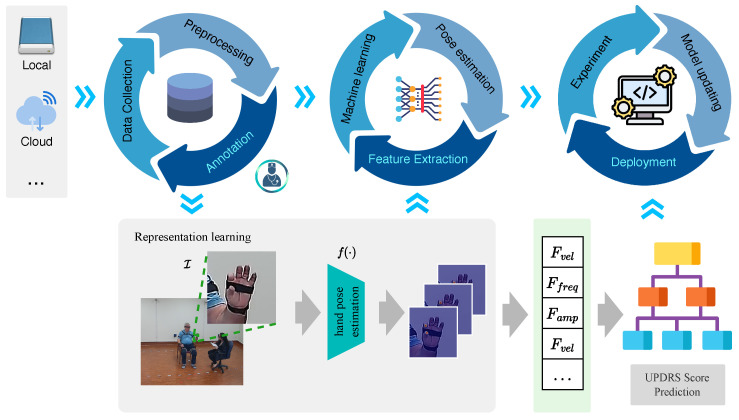
The main structure of the proposed framework. The pipeline consists of three distinct cycles: data cycle, model learning cycle, and algorithm deployment cycle.

Recognizing that data collection is a persistent and long-term pursuit, with our data repository steadily growing, we present a framework, illustrated in Figure 1, to maintain the lifecycle of the proposed system. Our framework consists of three sequential and independent cycles: a data cycle, a model learning cycle, and an algorithm deployment cycle.

### 3.1. Data Cycle

The data cycle consists of three parts; data collection, data preprocessing, and data annotation.

Data collection: Patient-generated videos can be sourced from diverse platforms, comprising self-recorded, home-based footage and professionally captured videos within laboratory settings by healthcare practitioners. This compilation may include a range of data forms: structured and unstructured data (encompassing text, images, and videos), as well as external data acquired via Application Programming Interfaces (APIs).

**Figure 2:**
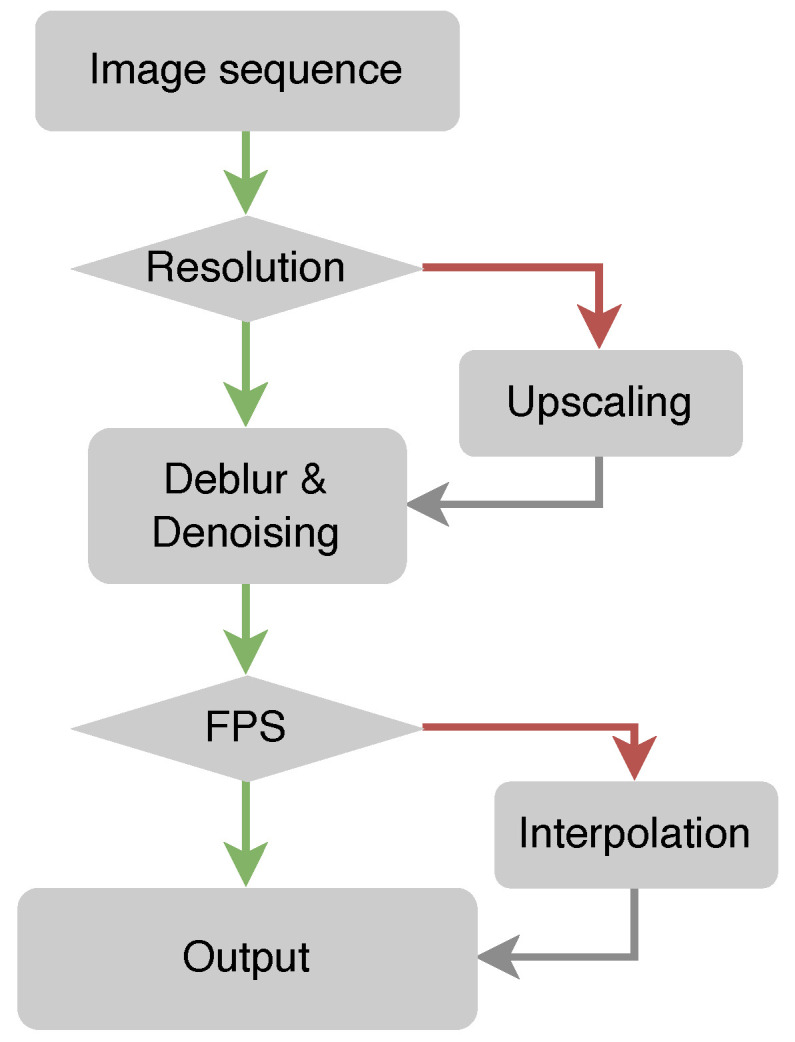
Data preprocessing flowchart.

Data preprocessing: Following data collection, the preprocessing phase is undertaken to transform the data into a format suitable for further analysis and model training. This phase includes handling missing values, eliminating outliers, normalizing or scaling features, and ensuring data format uniformity. We employed a range of methods to process the initial video data, as illustrated in Figure 2.

- Upscaling: Upscaling is employed to increase the resolution or dimensions of images or videos. This enhancement improves the quality of low-resolution data, thereby strengthening the model’s ability to extract significant features [41,42].
- Removing motion blur: Motion blur occurs in images or videos as a result of camera wobble or object motion [43]. When collecting multi-source data from various devices and environments, it is essential to remove motion blur to regain the sharpness and clarity of the data.
- Denoising: Noise may be introduced during the image- or video-acquisition process, leading to a degradation in quality and potentially hindering the capacity to extract meaningful features. A denoising step is often employed to reduce noise from the data while retaining the details.
- Frame interpolation: This technique generates additional frames in videos with low frame rates [44]. This process estimates the motion between existing frames and synthesizes new ones, thereby smoothing the motion and enhancing the temporal resolution.

These measures are designed to enhance data quality and facilitate the extraction of meaningful information for more-effective model training.

Data annotation: In this step, domain experts, typically clinicians, are involved in providing UPDRS scores or labels for the patient’s data, which serve as a reference for model training and evaluation [45]. It is imperative to establish precise and uniform guidelines for the scoring criteria in the annotation process. As shown in Table 1, the UPDRS documentation offers detailed instructions on how clinicians should allocate scores to patients’ data. Our study adopted a dual-annotator system. The primary annotator, an experienced Movement Disorder Specialist, annotated all the videos. In parallel, a secondary annotator, also skilled in movement disorders, was tasked with validation, acting as a quality control to guarantee the accuracy of the annotations. These annotators were blind to each other’s ratings throughout the process. To ascertain the annotations’ quality and reliability, we evaluated the inter-annotator agreement. It is essential to manage any discrepancies and disagreements among clinicians during the data-annotation process. This control is necessary to maintain the reliability and consistency of the annotated data.

In practice, the data cycle is iterative to allow the data repository to grow. As the data volume increases, this repetitive method facilitates continuous improvements and modifications to the models, leading to enhanced performance over time [45].

### 3.2. Model Learning Cycle

The model learning cycle consists of three key stages: hand pose estimation, feature extraction, and machine learning.

Initially, the 3D hand pose is extracted using the most-recent version of MediaPipe, updated in February 2023, to generate time series data of crucial joint positions [36]. MediaPipe was selected over other deep learning libraries for hand pose estimation due to its efficient performance in various recording contexts and its relatively lightweight architecture. The raw time series data of key joint positions are represented as $K=\{K_{j}\left(t\right)\}$, where *j* means the *j*-th key point, j=1,2,..,20, and *t* denotes the image frame index (for t=1,2,...,L). We calculated the time series of the thumb–index finger distance, denoted as I with length *L*, which corresponds to the distance between points $K_{4}$ and $K_{8}$ in the finger tapping task. In the second stage, we extracted 15 specific features for each video, as detailed in Table 3. These features encompass two demographic elements and 13 kinematic features, which were extracted through signal processing of the time series data. These kinematic features are aligned with the guidelines of the UPDRS instructions.

Lastly, based on these features, we trained a machine learning model.

#### 3.2.1. Time Series Preprocessing

**Figure 3:**
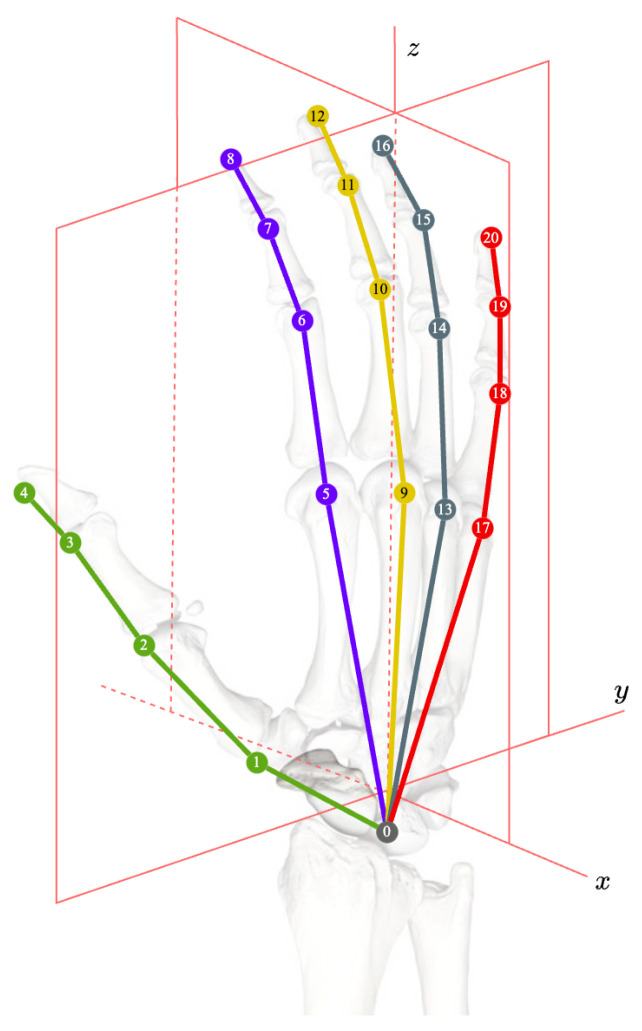
Illustration of the hand keypoints.

For the time series input I, $p=[p_{1},p_{2},...,p_{n},...,p_{N}]$ represents the peaks, $p_{n}$ represents the *n*-th peak, and $A_{n}$ represents the amplitude of the corresponding peak $p_{n}$. Given a hand model, as shown in Figure 3, $K_{j}=\left(k_{x,j},k_{y,j},k_{z,j}\right)$ represents the position of the *j*-th keypoint. The palm size at time *t* is estimated using $K_{0}$ and $K_{9}$ as:

(1) $$Palm_{t}=\sqrt{{\left(k_{x,0}-k_{x,9}\right)}^{2}+{\left(k_{y,0}-k_{y,9}\right)}^{2}+{\left(k_{z,0}-k_{z,9}\right)}^{2}}|t$$

Then, normalization is performed to make $Palm_{t}$ more suitable for subjectwise comparison, as below:

(2) $$I_{norm}=\frac{I}{Palm_{t}}$$

**Figure 4:**
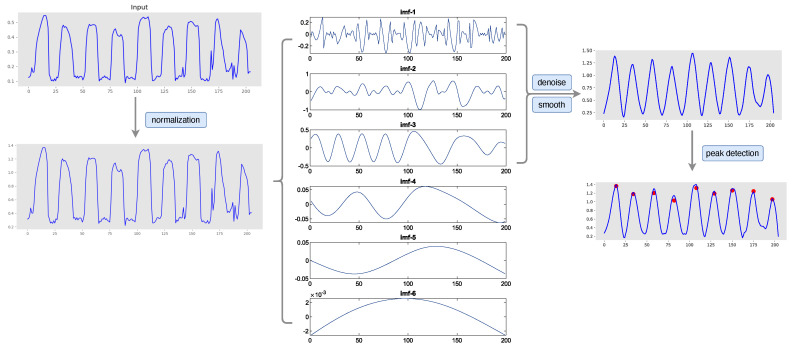
Depiction of the time series data preprocessing pipeline. The input time series, denoted as I, corresponds to the distance between $K_{4}$ and $K_{8}$ during the finger tapping task. The X−axis indicates the frame index within an image sequence, reflecting the duration of the finger tapping action. The Y−axis represents the normalized amplitude of finger movement, adjusted according to the palm size of each subject.

Following this, we applied Empirical Mode Decomposition (EMD) to decompose $I_{norm}$ into a series of Intrinsic Mode Functions (IMFs) and a residual component, as depicted in Figure 4. Each IMF captures a distinct oscillatory mode characterized by variable frequencies and amplitudes. In our approach, we selected the first three IMFs as the primary components of the signal, striking a balance between simplifying the data and preserving vital information. However, even after EMD-based decomposition, the signal may contain noise or unwanted artifacts, such as those arising from finger tremors. To address this, we employed a denoising and smoothing process to eliminate such disturbances.

After preprocessing and cleaning the time series data, the subsequent step involved the detection of peaks or specific events of interest within the signal. The peak-detection module was applied to find local maxima or minima, which served as key points in the data.

#### 3.2.2. Feature Extraction

**Feature vector: demographics**. In our model, we incorporated two demographic factors: age and sex. This inclusion was based on the observation that older individuals may exhibit signs of bradykinesia related to aging, independent of Parkinson’s Disease (PD), and that both age and sex can influence the progression of PD [1,46,47]. While the Unified Parkinson’s Disease Rating Scale (UPDRS) instructs raters to evaluate based on observable symptoms, there is a possibility that human annotators may subconsciously consider these demographic characteristics. Therefore, we chose to include age and sex as features in our model. Furthermore, given our goal of assessing the model’s explainability, incorporating these demographic factors may offer a more-comprehensive understanding that might not be immediately evident through the UPDRS guidelines alone.

**Feature vector: amplitude**. Based on the time series data, we extracted 5 amplitude-related features. First, according to Equations (3) and (4), we extracted $F_{amp-avg}$ and $F_{amp-var}$ as:

(3) $$F_{amp-avg}=\frac{\sum_{n=1}^{N}A_{n}}{N}$$

(4) $$F_{amp-var}=\frac{\sum_{n=1}^{N}{\left(A_{n}-F_{amp-avg}\right)}^{2}}{N}$$

**Figure 5:**
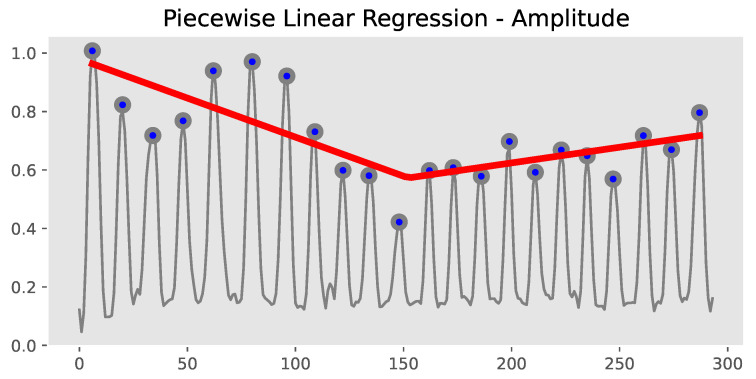
Extraction of $F_{amp-\alpha 1}$, $F_{amp-\alpha 2}$, and $F_{amp-bp}$.

Then, based on the peaks *P*, we applied piecewise linear regression to model the distinct patterns of different regions of the data, as shown in Figure 5:

(5) $$y=\alpha_{0}+F_{amp-\alpha 1}x+F_{amp-\alpha 2}\left(x-F_{amp-bp}\right)I\left(x>F_{amp-bp}\right)+\epsilon$$

where $\alpha_{0}$ is the intercept, $F_{amp-\alpha 1}$ and $F_{amp-\alpha 2}$ represent the slope coefficients of two segments, respectively, and ϵ represents the error term or residual, accounting for the deviations of the actual data points from the fitted model. I(·) is an indicator function that takes a value of 1 if *x* is greater than a specific breakpoint $F_{amp-bp}$ and 0 otherwise.

**Feature vector: velocity**. We extracted 5 velocity-related features. Here, we have

(6) $$V_{n}=\frac{A_{n}+A_{n-1}}{\Delta t_{n}}$$

where $\Delta t_{n}=t_{n}-t_{n-1}$ is the time interval between the *n*-th and (n−1)-th peak. Then, we have

(7) $$F_{vel-avg}=\frac{\sum_{n=1}^{N-1}V_{n}}{N-1}$$

(8) $$F_{vel-var}=\frac{\sum_{n=1}^{N-1}{\left(V_{n}-F_{vel-avg}\right)}^{2}}{N-1}$$

We also extracted another 3 features, $F_{vel-\alpha 1}$, $F_{vel-\alpha 2}$, and $F_{vel-bp}$, from the piecewise linear regression fitting of the velocity time series, to estimate the velocity trend.

(9) $$y=\alpha_{0}+F_{vel-\alpha 1}x+F_{vel-\alpha 2}\left(x-F_{vel-bp}\right)I\left(x>F_{vel-bp}\right)+\epsilon$$

**Feature vector: halt and hesitation**. In PD, “halts or hesitations” are characterized by brief instances of freezing or a delayed ability to initiate movements, often seen during repetitive actions. While the concept of the freezing of gait, essentially halts and hesitations during walking, is well-documented in PD [48], there is a scarcity of detailed definitions and quantitative assessments of “halts and hesitations” in upper limb movements during repetitive tasks.

**Figure 6:**
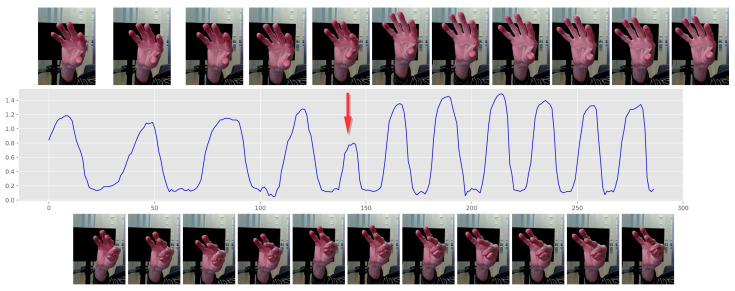
Illustration of halt and hesitation. The red row indicates the precise moment when the halt and hesitation occur.

Through extensive discussions to clarify “halts” and “hesitation” within our study, we defined “halts” as moments of complete stoppage, characterized by zero amplitude. In contrast, “hesitation” was identified as a noticeable reduction in velocity, registering values above zero, but significantly below a predefined empirical threshold. This distinction was quantitatively established in Equation (11), where the “hesitation” threshold was set at 20% of the average amplitude observed immediately before and after the occurrence. More specifically, we define the “halt and hesitation” in finger tapping tasks as follows: given a time series of the distance between the keypoints $K_{4}$ and $K_{8}$, $p=[p_{1},p_{2},...,p_{n},...,p_{N}]$ represents the detected peaks. A curve fitting was applied to p, aiming to estimate the trend of the peaks. Assuming $p_{n}$ to be the peak when halt and hesitation happened (the red arrow in Figure 6), ${\hat{p}}_{n}$ represents the predicted peak based on the fitted model. The residual between the observed value and the predicted value is denoted by $|{\hat{p}}_{n}-p_{n}|$. Then, the feature “halt and hesitation” could be defined as:

(10) $$F_{hh}\equiv \begin{array}{cc} 1\; & \mathrm{if}\;|{\hat{p}}_{n}-p_{n}|\;\geq \theta \;\\ 0\; & \mathrm{if}\;|{\hat{p}}_{n}-p_{n}|\;<\theta \;. \end{array}$$

As demonstrated in Figure 6, in this situation, the patient may experience a sudden interruption or drop in his/her ability to perform repetitive finger tapping movements. The threshold θ is defined as:

(11) $$\theta =\alpha \cdot \frac{A_{n+1}+A_{n-1}}{2}$$

where α is a parameter controlling the detection threshold. In our framework, we empirically set α to 0.2.

#### 3.2.3. Machine Learning

We chose the Decision Tree approach as our primary classification method to improve the interpretability and quantification of assessments tailored to our specific task. Decision Trees offer clear interpretability and the ability to visually represent the model, facilitating an uncomplicated explanation of the decision-making process. Inherently proficient in managing categorical data, Decision Tree models are particularly suitable for modeling ordinal UPDRS scores. Additionally, the inherent transparency and interpretability of Decision Tree models correspond well with the explicit criteria set forth by the UPDRS. This alignment ensures that our model’s predictions are not only easily understandable, but also hold clinical relevance.

**Figure 7:**
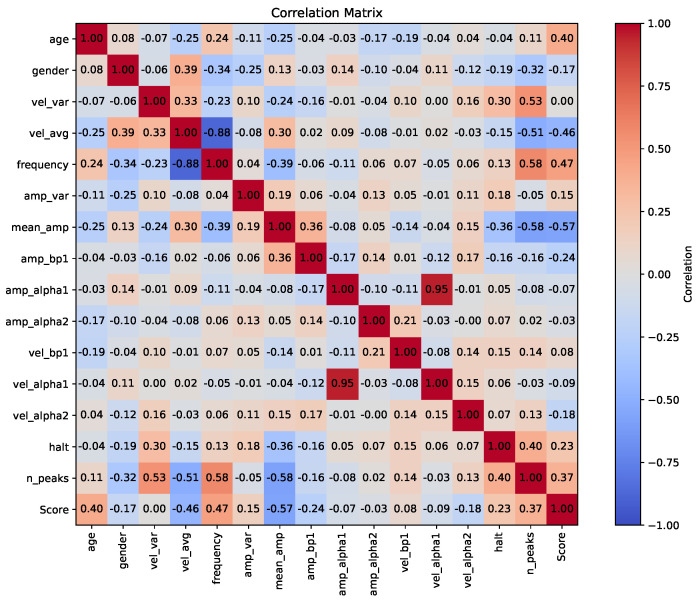
Correlations between the 15 extracted features and UPDRS scores.

The Pearson correlation coefficients between the 15 features and the UPDRS scores are presented in Figure 7. Redundant or overlapping features can lead to inefficient splits and diminished predictive capability in the model. However, as we identified a high correlation solely between the frequency and velocity of tapping, we decided to maintain all 15 original features in our analysis. This approach was chosen to ensure that the unique contribution of each feature is preserved.

### 3.3. Algorithm Deployment Cycle

Model experimentation: This initial phase involved the training and evaluation of various models using different algorithms, hyperparameters, and subsets of data. The purpose of model experimentation is to identify the most-promising model based on its performance across various evaluation metrics.

Model updating: After selecting a suitable model, further refinement and improvement are often necessary. This updating process includes retraining the chosen model with additional data or altered hyperparameters to boost its performance. The goal of this iterative procedure is to enhance the model’s accuracy, robustness, and ability to generalize.

Deployment: The deployment phase involves making the refined and updated model accessible in a production setting. This stage encompasses establishing the required infrastructure to host and operate the model, which may involve servers, cloud services, or containerization platforms. Additionally, it includes the development of the necessary APIs or interfaces to enable the user or system access to the model for predictions or analyses.

The deployment cycle in MLOps begins with model experimentation, where a variety of models are trained and assessed. Following the selection of the most-effective model, it enters the updating stage for further improvement. The enhanced model is then deployed in a production environment, ready for practical application.

It is crucial to recognize that this deployment cycle is often iterative and ongoing. As new data are introduced, models require updates and redeployment to meet shifting demands. Continuous monitoring, testing, and feedback are integral to the deployment cycle, ensuring the deployed model maintains its performance, reliability, and efficacy in real-world settings.

## 4. Experiments

### 4.1. Participants and Dataset

RGB, single-view videos were collected in two settings with different camera and supervision setups: in-lab and in-clinic. In the laboratory setting, patients were invited to perform the finger tapping task as outlined in the MDS-UPDRS guidelines. This task was carried out in a spacious laboratory room. A research assistant was present to guide the patients through the task, which was captured by eight cameras (Lorex 4K Bullet IP Camera/E841CA-E from Lorex Technology Inc., Toronto, ON, Canada ) mounted on the walls. The raw data from these cameras had a resolution of 3840×2160 px and a frame rate of 15/s. Of the eight camera angles, our primary focus was on the single camera view that provided the clearest, most-unobstructed perspective of the hand movements. While not a central aspect of this study, an inertial sensor was also attached to the patient’s hand during recording, visible as a black band on the palm in Figure 1. In the clinic setting, patients were recruited to perform various tasks from the MDS-UPDRS, including finger tapping, in a designated room within the waiting area of the Movement Disorders Clinic. These tasks were recorded using an AI camera, OAK-D (Luxonis, Littleton, CO, USA), installed on an iMac 24” desktop (Apple, Cupertino, CA, USA). In this setting, patients received little or no assistance from a research assistant in using the app for recording. The raw RGB data collected in the clinic had a resolution of 1920×1080 px and a frame rate of 60 fps.

A total of 65 patients diagnosed with PD were recruited to do the UDPRS assessment. The clinical diagnosis for each patient was confirmed based on the United Kingdom Parkinson’s Disease Society Brain Bank clinical diagnostic criteria. To ensure the quality of the data, specialists manually inspected the videos, applying several criteria to evaluate their suitability. Videos were excluded if they exhibited poor visibility of motion, such as the hands frequently moving out of the camera frame, a significant distance between the hands and the camera, inconsistent execution of tasks, or frequent interruptions (verbal or physical) either by the patients or bystanders. After the exclusion process based on these quality criteria, 75 videos from 50 patients were deemed suitable for assessing the effectiveness of the proposed method. This dataset comprised 20 subjects recorded in a laboratory setting and 30 subjects in a clinic environment, as detailed in Table 4. For the purposes of our study, 60 videos were designated for the training set and 15 videos for the testing set. The selection of testing subjects was randomized, ensuring a balanced representation of different categories. The Research Ethics Board of the University of British Columbia approved this study, and all participants provided written consent. All individuals were evaluated using the MDS-UPDRS, with the entire evaluation process captured on video. Due to regulations safeguarding patient privacy, these videos are not permitted to be made publicly available.

### 4.2. Evaluation Matrix

To evaluate the performance of our model, we employed well-known metrics such as accuracy, precision, recall, and the F1-score. These metrics are defined as follows:

(12) $$\begin{array}{cc} Accuracy & =\frac{TP+TN}{TP+FN+FP+TN} \end{array}$$

(13) $$\begin{array}{cc} Precision & =\frac{TP}{TP+FP} \end{array}$$

(14) $$\begin{array}{cc} Recall & =\frac{TP}{TP+FN} \end{array}$$

where TP,TN,FP, and FN represent “true positive”, “true negative”, “false positive”, and “false negative”, respectively, and the F score (F1 when β = 1 and F2 when β = 2) is defined as:

(15) $$F_{\beta }=\left(1+\beta^{2}\right)\cdot \frac{\;\mathrm{Precision}\;\cdot \;\mathrm{Recall}\;}{\beta^{2}\cdot \;\mathrm{Precision}\;+\;\mathrm{Recall}\;}$$

In our evaluation matrix for accuracy, we considered two scenarios:

- $y_{pred}$ = $y_{gt}$, which is reported as “Accuracy(t1)”, represents that the prediction $y_{pred}$ is equal to the ground truth $y_{gt}$.
- $y_{pred}\in \left[y_{gt}-1,y_{gt},y_{gt}+1\right]$, reported as “Accuracy(t2)”, represents that the prediction is within a range of the ground truth, which could also be called “acceptable” accuracy. This situation takes the inter-annotator agreement into consideration, and we will further analyze this situation in the result part.

### 4.3. Method for Comparison

To assess the performance of our proposed method, we compared it against two recent studies: AEMPD-FCN [49] and FTTST [23]. It is important to note that there might be significant differences in data quality, volume, and time series length between our study and those in the referenced work, which could potentially influence the comparative results. Additionally, we explored the effectiveness of different classifiers by including results from three other methods. These methods all employ the same feature-extraction framework, but differ in their classification approaches, such as Support Vector Machine (SVM) and Random Forest (RF). Specifically, baseline-SP is the baseline model that follows the UDPRS criteria precisely. To some extent, this criterion is overly strict for some data points situated at the boundaries. For example, a slight decrease in amplitude or velocity could be classified as decrementing features. *F*15-SVM-GA uses the 15 features as the input vector to the SVM classifier, where the Genetic Algorithm is used to search for the best parameters. *F*15-RF uses Random Forest for classification, and in *F*15-DT, the DT represents a Decision Tree.

## 5. Results and Discussion

### 5.1. Labeling UPDRS Finger Tapping Videos

In our study, we calculated the level of agreement between annotators, as shown in Table 5. “Complete”: This term refers to the percentage of instances where both annotators assigned an identical score to a specific piece of data. “Acceptable”: This category covers cases where the scores given by the two annotators differed by either 0 or 1. It represents a more-lenient agreement threshold. “None”: This indicates the percentage of cases where the discrepancy in scoring between the two annotators was more than 1. The data presented in Table 5 highlights the inherent difficulty in achieving complete consensus among human annotators, even when they are experts in their field, as has been previously observed [50]. Acknowledging this challenge, we report two types of accuracy metrics in our experiments, namely Accuracy(t1) and Accuracy(t2), as defined in Section 4.2. These metrics are intended to evaluate the efficacy of our proposed method, taking into account the varying levels of agreement among annotators.

### 5.2. Classification of UPDRS Finger Tapping Score

**Figure 8:**
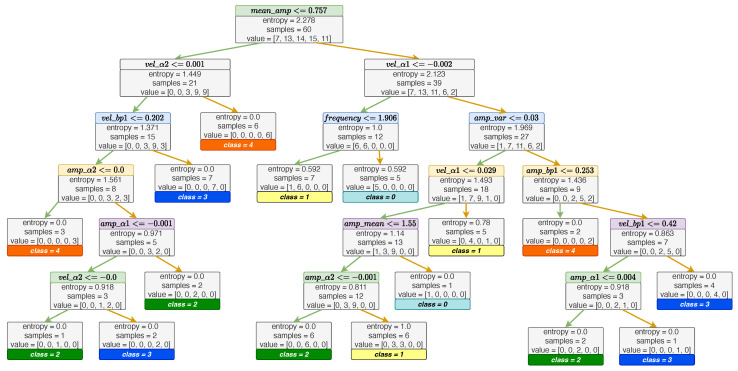
Structure of the proposed Decision Tree.

**Figure 9:**
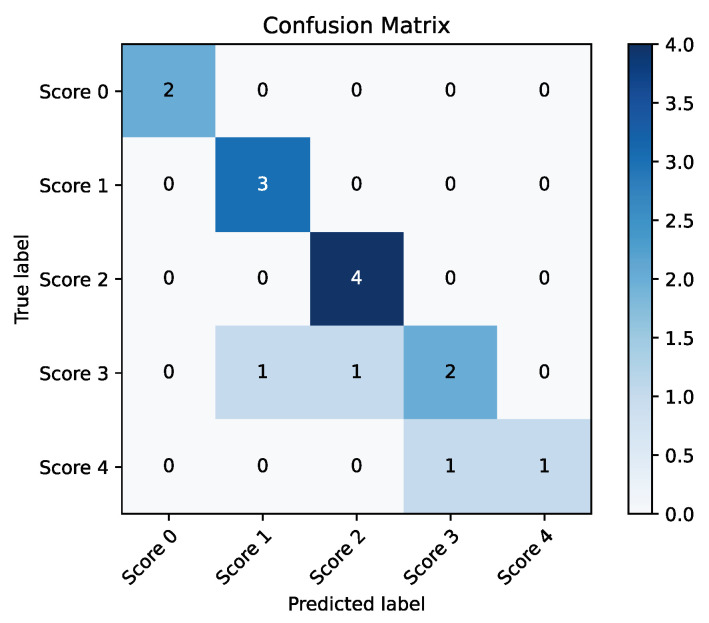
Confusion matrix of the predicted score labels and the true labels.

First, the main classification task was performed on the finger tapping features using the clinical ratings. The proposed method achieved a high accuracy rate, an Accuracy(t1) of 80% and an Accuracy(t2) of 93.3%. Table 6 lists the specific accuracy(t1 and t2), precision, recall, and F-score of the final prediction results on five UPDRS scores. The structure of the Decision Tree and the confusion matrix of the classification results are shown in Figure 8 and Figure 9, respectively.

**Clinical interpretation:** Overall, the Decision Tree our model produced (as shown in (Figure 8)) aligns well with the MDS-UPDRS rating guidelines. These guidelines instruct raters to evaluate finger tapping based on: (i) amplitude, (ii) velocity, (iii) decrement in amplitude or velocity during repetitive tapping, and (iv) the presence of halts or hesitations [3]. The root node in our Decision Tree (Node 0) utilizes the “mean amplitude” feature, dividing the data into two paths and indicating amplitude as a pivotal factor in the finger tapping assessment. In the second layer, the nodes “vel_alpha2” and “vel_alpha1” reflect the velocity decrement, suggesting that greater decrements often correspond with lower UPDRS scores. This points to the relevance of the “decrement in amplitude or velocity” criterion in cases of mild bradykinesia, while in more-severe cases, the velocity notably reduces from the beginning of the task. Further nodes incorporate amplitude, velocity, and decrement aspects, aligning conceptually with the MDS-UPDRS guidelines, highlighting the model’s ability to offer more-quantifiable data and reduce intra-rater variability compared to human ratings.

Additional clinical insights emerged from the Decision Tree. Notably, “halts and hesitations” had minimal impact on the model. This could be due to the rarity and lesser correlation of upper limb freezing with PD severity compared to gait freezing. Our model’s interpretation suggests a non-linear relationship between these halts and overall bradykinesia severity [51]. It is important to recognize the limitations posed by the relatively small video dataset used for the AI training, potentially under-representing upper limb freezing episodes Furthermore, our study’s definition of halts and hesitations, based purely on significant amplitude reduction without a time duration criterion, may not fully align with clinical descriptions of a halt as a prolonged pause. Future studies, especially with larger datasets, might need a more-refined definition, possibly incorporating time-based thresholds for a better characterization of these phenomena. Lastly, while we considered demographic variables like age and gender, their absence in the Decision Tree underscores that UPDRS ratings focus on motor performance irrespective of demographic factors.

## 6. Limitations and Future Work

Our study marks a step forward in automated Parkinson’s Disease (PD) symptom assessment, yet it is important to recognize its limitations. Firstly, the limited number of patient videos used for training the model, though yielding promising accuracy, may not fully represent the broad spectrum of PD symptoms, including pronounced halts or hesitation phenomena. A more-extensive dataset will be critical for enhancing the model’s generalizability and robustness. Secondly, the study’s focus was solely on the finger tapping task, an important, but not complete aspect of bradykinesia evaluation. Other key tasks in assessing bradykinesia, such as hand movements and leg agility, were not included. Furthermore, PD involves a variety of motor symptoms beyond bradykinesia. Future research will aim to extend our video-based approach to cover a broader range of motor tasks from the UPDRS, enabling a more-comprehensive assessment of PD motor symptoms. Thirdly, the study’s setting was restricted to controlled clinical environments, which may not fully replicate the varied conditions in which the model might be used. Factors like changing lighting, patient positioning, and camera quality or angles, crucial for video quality, were not thoroughly examined. Additionally, our approach of manually excluding low-quality videos needs to be replaced by automated algorithms in future work. These algorithms should be capable of distinguishing between low- and high-quality videos based on predefined quality thresholds. Finally, while not directly related to our scientific methodology, the ethical aspects of patient privacy and data security are crucial. As we move towards real-world applications of this technology, a multidisciplinary approach involving legal, ethical, clinical, engineering, and patient stakeholders is essential. This collaborative effort is key to addressing the complex ethical landscape, ensuring the protection of patient privacy and data security.

## 7. Conclusions

The proposed innovative vision-based classification model offers a promising solution to automate UPDRS scoring accurately during the finger tapping test, a crucial aspect of motor evaluation in Parkinson’s Disease (PD). Our approach adheres to the UPDRS guidelines by extracting the relevant features and quantifying them, enabling a thorough analysis. The proposed method was designed to adapt to future changes in patient demographics, disease subtypes, and technological advancements. We are actively working to expand our data collection using a variety of devices and in different settings. This effort is crucial to enhance the model’s applicability and effectiveness across diverse contexts and technological platforms.

Our comprehensive experiments demonstrated the effectiveness and reliability of the proposed method, producing robust results with datasets obtained both in laboratory and clinical settings.

## Figures and Tables

**Table 1:** MDS-UDPRS criteria for the finger tapping task.

UPDRS Score	Symptoms
0: Normal	No problems.
1: Slight	Any of the following:
	a. one or two interruptions or hesitations;
	b. slight slowing;
	c. the amplitude decrements near the end.
2: Mild	Any of the following:
	a. 3 to 5 interruptions during tapping;
	b. mild slowing;
	c. the amplitude decrements midway in the 10-tap sequence.
3: Moderate	Any of the following:
	a. more than 5 interruptions or at least one longer arrest (freeze) in ongoing movement;
	b. moderate slowing;
	c. the amplitude decrements starting after the 1st tap.
4: Severe	Cannot or can only barely perform the task because of slowing, interruptions, or decrements.

**Table 2:** Overview of selected studies on video-based bradykinesia assessment in Parkinson’s disease: “Input” specifies the type of video format utilized; “Pose Estimation” indicates the software library employed for human pose analysis; “Extracted Features” pertains to the kinematic attributes obtained from the pose estimation data over time; “Method” details the specific machine learning approach used for categorizing the data; “Result” summarizes the outcomes of the classification process; “Tasks” outlines the specific UPDRS activities evaluated in each study.

Authors, Year [Ref]	Input	Pose Estimation	Extracted Features	Method	Results	Tasks
Z. Guo et al., 2022 [23]	RGB–depth	A2J	Amplitude, velocity	SVM	Severity level	Finger tapping
Morinan et al., 2023 [4]	RGB	Openpose	11 features representing speed, amplitude, hesitations, and decrement	Random Forest	Binary/ordinal classification	Finger tapping
Sarapata et al., 2023 [30]	RGB	Openpose	Region of interest extraction of motor tasks, followed by kinetic feature extraction for each task	Random Forest	Binary/ordinal classification	Finger tapping, hand movement, pronation/supination, toe tapping, leg agility, rising from a chair, gait
Islam et al., 2023 [31]	RGB	MediaPipe	53 features representing speed, amplitude, hesitations, and decrement	LightGBM	Regression	Finger tapping
Proposed method	RGB	MediaPipe	15 features representing demographics, amplitude, velocity, halt and hesitations, and decrement	Decision Tree	Severity level	Finger tapping

**Table 3:** Fifteen features extracted from the finger tapping time series.

Features	Description
$F_{age}$	Age of the patients
$F_{gen}$	Gender of the patients
$F_{amp-var}$	Variance of the amplitude
$F_{amp-avg}$	Mean of the amplitude
$F_{amp-bp}$	Breakpoint of the amplitude
$F_{amp-\alpha 1}$	Slope 1 of the amplitude
$F_{amp-\alpha 2}$	Slope 2 of the amplitude
$F_{vel-var}$	Variance of the velocity
$F_{vel-avg}$	Mean velocity of the finger movement
$F_{vel-bp}$	Breakpoint of the velocity
$F_{vel-\alpha 1}$	Slope 1 of the velocity
$F_{vel-\alpha 2}$	Slope 2 of the velocity
$F_{freq}$	Frequency of finger tapping
$F_{hh}$	Number of halts and hesitations
$F_{p}$	Number of peaks of the time series

**Table 4:** Baseline characteristics of the participants (n = 50) and the videos (n = 75).

Baseline Characteristics	
Age, years	
Median	71
Mean (SD)	69.65
Sex	
Female/Male, n/n (%/%)	19/31(38/62)
Need assistance for walking, n (%)	2 (4)
Dominant side of Parkinsonism	
Left	23
Right	27
UPDRS Score	
0/1/2/3/4, n	8/21/18/17/11

**Table 5:** Inter-annotator agreement analysis in the proposed task.

Complete	Acceptable	None
52%	97%	3%

**Table 6:** Performance results of UPDRS classification using different classifiers.

Method	Evaluation Metric				
	**Accuracy(t1)**	**Accuracy(t2)**	**Precision**	**Recall**	**F1**
AEMPD-FCN [49]	0.267	0.60	0.18	0.19	0.173
FTTST [23]	0.467	0.80	0.50	0.60	0.513
baseline-SP	0.467	0.733	0.480	0.60	0.509
F15-SVM-GA	0.533	0.867	0.553	0.590	0.556
*F*15-RF	0.533	0.867	0.533	0.552	0.540
F15-DT	0.80	0.933	0.843	0.80	0.797

## Data Availability

The data are not allowed to be made public according to the relevant regulations protecting patient privacy.
